# Early Pregnancy Body Roundness Index and Development of Adverse Pregnancy Outcomes: A Secondary Analysis of the nuMoM2b Study

**DOI:** 10.1002/oby.70274

**Published:** 2026-08-05

**Authors:** Kelsey J. Pape, Taylor M. Bynarowicz, Sruthi Bhamidipalli, Yan Tong, Maisa N. Feghali, David M. Haas, Uma M. Reddy, Robert M. Silver, Lynn M. Yee, Christina M. Scifres

**Affiliations:** ^1^ Department of Obstetrics & Gynecology, Division of Maternal‐Fetal Medicine Indiana University School of Medicine Indianapolis Indiana USA; ^2^ Department of Obstetrics & Gynecology Indiana University School of Medicine Indianapolis Indiana USA; ^3^ Department of Biostatistics Indiana University School of Medicine Indianapolis Indiana USA; ^4^ Department of Obstetrics & Gynecology, Division of Maternal‐Fetal Medicine University of Pittsburgh Medical Center Magee‐Womens Hospital Pittsburgh Pennsylvania USA; ^5^ Department of Obstetrics & Gynecology, Division of Maternal‐Fetal Medicine Columbia University Vagelos College of Physicians and Surgeons New York New York USA; ^6^ Department of Obstetrics & Gynecology, Division of Maternal‐Fetal Medicine University of Utah School of Medicine Salt Lake City Utah USA; ^7^ Department of Obstetrics & Gynecology, Division of Maternal‐Fetal Medicine Northwestern University Feinberg School of Medicine Chicago Illinois USA

**Keywords:** body composition, gestational BMI, obesity, risk factors, waist circumference

## Abstract

**Objective:**

This study assessed the association between early pregnancy body roundness index (BRI) and adverse pregnancy outcomes in nulliparas.

**Methods:**

In this secondary analysis of a multicenter prospective cohort study, waist circumference, height, and weight were measured between 6 and 13 6/7 weeks' gestation and used to calculate BMI and BRI (364.2–365.5 × {1 − [(waist circumference/2π)/(0.5 × height)]^2^}^0.5^). Four BRI and BMI quantiles (Q1‐Q4) were defined, and rates of adverse pregnancy outcomes were compared using multivariable logistic regression.

**Results:**

Among 9671 individuals, greater BRI was associated with gestational diabetes (GDM), hypertensive disorders, preterm birth, cesarean delivery, and large‐for‐gestational age infants. Discordant BRI and BMI assignments affected GDM and cesarean odds. Those with Q2 BMI but Q3–Q4 BRI had increased odds of GDM (aOR 2.28, 1.49–3.47) and cesarean (aOR 1.39, 1.15–1.68). Those with Q3 BMI and Q1–Q2 BRI demonstrated reduced odds for GDM (aOR 0.31, 0.15–0.63) and cesarean (aOR 0.75, 0.60–0.94). Participants with Q4 BMI and Q1–Q3 BRI experienced decreased odds for cesarean (aOR 0.55, 0.43–0.70) but similar GDM odds.

**Conclusions:**

Greater first trimester BRI, a reflection of central adiposity, is associated with adverse pregnancy outcomes. Early pregnancy BRI may improve risk stratification for GDM and cesarean delivery beyond BMI.

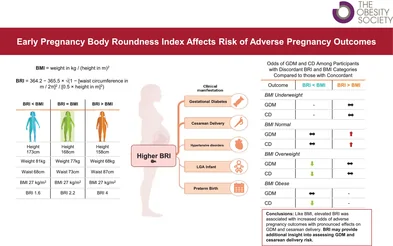

## Study Importance

- What is already known?
  ○ The effects of excess body weight measured by BMI early in pregnancy are well described, but the impact of central adiposity independent of BMI is less clear. Prior studies on BRI in pregnancy vary in gestational age of measurement and outcomes assessed.
  ○ BRI is associated with adverse cardiovascular outcomes and all‐cause mortality in non‐pregnant adults. Comprehensive data on its association with adverse pregnancy outcomes are limited.
- What does this study add?
  ○ Our secondary analysis of a large, multicenter prospective cohort using first trimester BRI identified dose‐dependent increased odds of nearly all adverse pregnancy outcomes assessed, similar to BMI.
  ○ Early pregnancy BRI had a stronger association with gestational diabetes (GDM) and cesarean delivery than BMI. Moreover, BRI exerted differential effects, particularly for GDM and cesarean delivery, on participants with BRI values discordant from assigned BMI category.
- How might these results change the direction of research or the focus of clinical practice?
  ○ GDM and cesarean delivery carry significant immediate and future health consequences. Improved understanding of those at highest risk facilitates individualized preconception counseling, promotion of health optimization prior to pregnancy, and defining of best practices for obstetric care.
  ○ Although our study indicates BRI may not provide broad benefit beyond BMI in pregnancy, BRI is noninvasive and easily obtained. Future studies can expand upon our results by clarifying and evaluating BRI thresholds and assessing interventions directed toward mitigating GDM and cesarean delivery risk in those with elevated BRI.

## Graphical abstract

Greater body roundness index is associated with increased odds of adverse pregnancy outcomes. Thomas et al. Relationships between body roundness with body fat and visceral adipose tissue emerging from a new geometrical model. Obesity (Silver Spring). 2013 November; 21(11): 2264–2271. Central image created with BioRender.

## 1 Introduction

Obesity rates are rising globally [1], and obesity is becoming more common during pregnancy. The 2017–2018 National Health and Nutrition Examination Survey (NHANES) identified that nearly one in three US adults are living with overweight or obesity [2]. By 2030, approximately 50% of US adults will have obesity [1]. The risks of obesity in pregnancy include gestational diabetes (GDM), hypertensive disorders of pregnancy, cesarean delivery, large‐for‐gestational‐age (LGA) birth weight, stillbirth, NICU admission, and childhood risk of cardiometabolic disorders [3, 4].

Obesity has traditionally been defined using body mass index (BMI), which is limited in its ability to phenotype weight and adiposity as it cannot account for lean mass. A multinational group of experts recently suggested that direct measurement of body fat or anthropometric criterion be used in addition to BMI to define clinical obesity [5]. Several anthropometric indices have been evaluated in predicting long‐term health consequences of excess adiposity. The body roundness index (BRI), which ranges from 1 to 16, was created by Thomas et al. [6] as a geometric index to estimate visceral adiposity with natural waist circumference and height. BRI may offer higher prediction of visceral and body fat than waist circumference alone. In non‐pregnant populations, BRI outperformed BMI for predicting metabolic syndrome, prediabetes, and diabetes in some studies [7, 8]. However, this was not the case for hypertension [9]. Greater BRI has recently been found to be associated with cardiovascular morbidity and all‐cause mortality [10, 11, 12].

While prior studies have measured the impact of anthropometric indices on adverse perinatal outcomes, most focus on a single outcome and results are mixed [13]. Shi et al. [14] recently found that greater BRI was associated with a patient‐reported history of GDM in an analysis of 3343 non‐pregnant women using NHANES data. BRI also demonstrated better predictive capacity compared to BMI by receiver operating characteristic (ROC) curve analysis. In small cohort studies, elevated BRI was a risk factor for GDM, fetal macrosomia, and increased insulin resistance and inflammatory markers [15, 16].

There is a paucity of data evaluating the relationship between BRI and additional perinatal outcomes, particularly those interrelated with GDM, such as cesarean delivery, hypertensive disorders of pregnancy, and LGA birth weight [15]. In addition, recent data suggest women have higher BRI values than men [12], and that BRI has a stronger association with adverse cardiometabolic outcomes in females compared to males, highlighting the relevance of exploring this measure in pregnancy [17]. We therefore evaluated the association between early pregnancy BRI and adverse perinatal outcomes in a diverse contemporary cohort of nulliparous pregnant patients in the US. Additionally, we compared BRI's association with these adverse outcomes to that of BMI.

## 2 Methods

This is a secondary analysis of the nuMoM2b (Nulliparous Pregnancy Outcomes Study: Monitoring Mothers‐to‐be) Study. The protocol for the nuMoM2b study has been published previously [18]. Briefly, the nuMoM2b study was a prospective multicenter cohort study of 10,038 pregnant individuals with singleton gestations recruited from eight clinical sites in the US. The IRBs at participating centers approved of the nuMoM2b study, and participants were consented for both the parent study and future analyses. Clinical and demographic characteristics were obtained from interviews, medical record reviews, and in‐person clinical measurements. At study visit 1 during the first trimester (between 6 0/7 and 13 6/7 weeks' gestation) the following relevant characteristics were measured: height, weight, and natural waist circumference. Waist circumference was measured using the National Institutes of Health protocol: in a standing position, a flexible tape was used to obtain duplicate measurements with the bottom edge of the tape at the top of the right iliac crest at the end of a normal exhalation [18]. First trimester BMI was calculated as weight in kilograms divided by height in meters squared. The uterus does not become an abdominal organ until the second trimester after which it may impact waist circumference validity [19]. BRI was calculated using the following [6]:

$$364.2-365.5\times \sqrt{1-{\left(\frac{\text{Waist circumference}}{\pi \times \text{Height}}\right)}^{2}}$$

Additional participant demographics were recorded, including maternal age, marital status, insurance type, household income, chronic conditions such as hypertension and pre‐existing diabetes, and self‐reported alcohol or tobacco use in the previous month. Race and ethnicity were self‐reported and characterized as non‐Hispanic White, non‐Hispanic Black, Hispanic, Asian, multiracial, Native Hawaiian, and American Indian; those in the last three categories and those who had identities other than those listed were combined for analysis to avoid reporting on small counts. Race and ethnicity were reported to describe the demographic characteristics of the cohort and assess generalizability.

At least 30 days after delivery, trained and certified abstractors reviewed the medical records of all participants and their neonates to track final birth outcomes. Maternal outcomes for this analysis included gestational weight gain, cesarean delivery, GDM, and hypertensive disorders of pregnancy, which included gestational hypertension, pre‐eclampsia, or eclampsia [20]. To account for the impact of pregnancy duration on gestational weight gain, *z*‐scores were calculated using published weight‐gain‐for‐gestational‐age reference charts for each BMI category [21]. GDM diagnosis was based on medical record review and testing was performed according to each site's local protocol (of the GDM test results available, 92.8% used two‐step testing with a non‐fasting 50 g glucose challenge test [OGTT] followed by a fasting 100 g oral glucose test if the initial screen was positive and 7.2% were a 75 g OGTT). Neonatal outcomes for this analysis included preterm birth, stillbirth, small‐for‐gestational‐age (SGA) birth weight, and large‐for‐gestational‐age (LGA) birth weight. Preterm birth was defined as a delivery prior to 37 0/7 weeks and included both spontaneous and indicated deliveries. SGA and LGA were defined using a US birth weight reference corrected for implausible gestational age estimates and assigned based on newborn sex [22].

Participants were excluded from the analysis if they had missing values for weight, height, or waist circumference at study visit 1, which are needed to calculate first trimester BRI and BMI. Previous studies have used varied grouping methods and reference comparators to evaluate outcomes between BRI categories [12, 14]. Given that our main goal was to evaluate BRI's impact on pregnancy and performance compared to BMI, we opted to use the definitions of BMI to anchor our analysis. Therefore, we identified the percentile distribution of BMI categories: ≤ 3rd (“underweight”), 4th–52nd (“normal”), 53rd–77th (“overweight”), and ≥ 78th (“obesity”) percentiles, respectively [23]. We then used these percentiles to create four corresponding quantiles of BRI (Q1, lowest to Q4, highest). The second quantile (Q2, BRI 1.68–3.20) was selected as the reference group for respective analyses.

This report follows the Strengthening the Reporting of Observational Studies in Epidemiology (STROBE) guidelines [24]. Maternal baseline demographics and perinatal outcomes were compared between first trimester BRI or BMI categories, respectively, using the Chi‐squared test, Fisher's exact test, or ANOVA, as appropriate. Multivariable logistic regression models were fitted to assess the associations of each adverse outcome with early pregnancy BRI or BMI separately. Covariates included in the multivariable logistic regression analyses were selected based on biologic plausibility or socio‐structural impact on the outcomes, including maternal age, race, insurance type, current tobacco use, pregestational diabetes, and chronic hypertension. Race was included in the multivariable models as a marker for the structural racism that may impact health outcomes. ROC curves for early pregnancy BRI and BMI as continuous variables were constructed using the predictive values derived from the respective multivariable logistic regression models for each outcome. AUC values from the ROC curves were compared to assess the relative discriminatory ability of first trimester BRI and BMI for each outcome. Finally, individuals were categorized into three groups based on the concordance or discordance between their first trimester BRI and BMI classifications: BRI < BMI (a lower quantile ranking in BRI than BMI), BRI = BMI (concordant quantile ranking between BRI and BMI), and BRI > BMI (a higher quantile ranking in BRI than BMI). For example, a person who has a Q3 BRI (3.21–4.45) but who has a BMI in the obesity category (Q4) would be classified in the BRI < BMI group.

Stratified by BMI classification, multivariable logistic regression models were fitted within each early pregnancy BMI category to compare the risk of each perinatal outcome between the BRI < BMI group or BRI > BMI group with the BRI = BMI group (the reference category). All statistical analyses were performed using SAS 9.4 (SAS Institute Inc., Cary, NC).

## 3 Results

**FIGURE 1:**
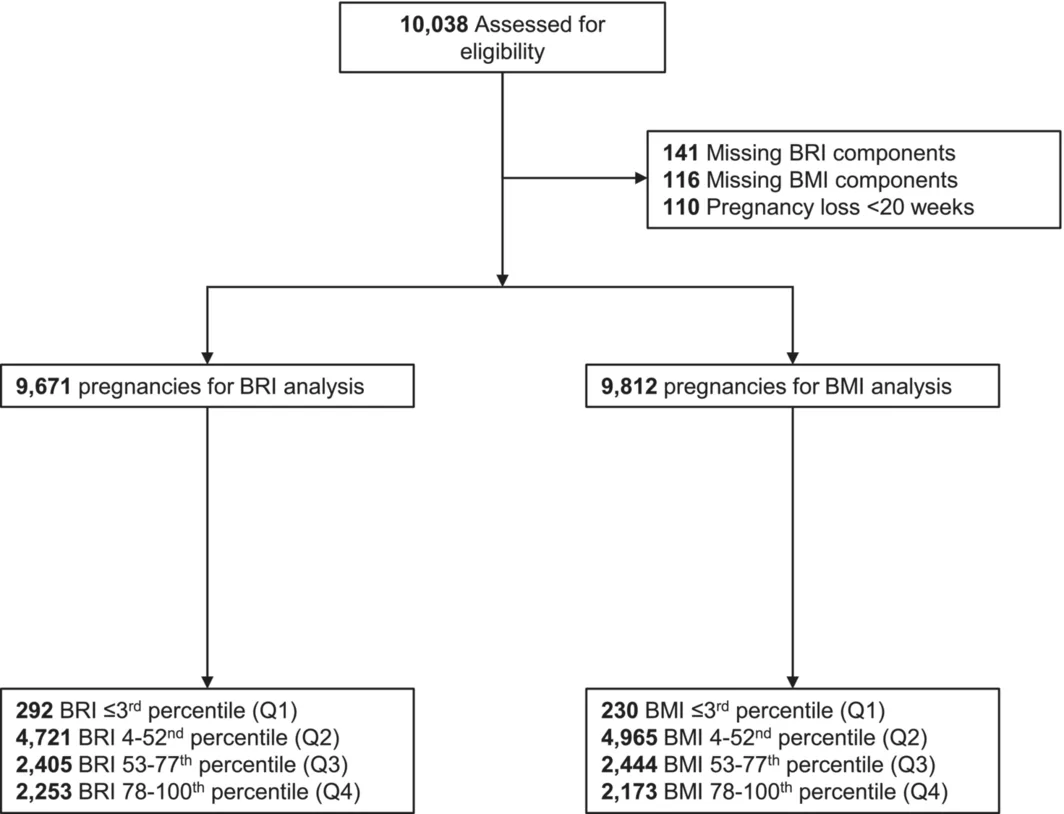
Participant flow diagram.

The parent study enrolled 10,038 nulliparous pregnancies from October 2010 to May 2013. Over 60% of participants enrolled and had anthropometric measures completed at ≥ 12 weeks' gestation, 27% enrolled between 10 and 12 weeks, and 10% between 8 and 10 weeks. Less than 2% enrolled prior to 8 weeks [18]. One hundred and ten participants were excluded due to pregnancy loss under 20 weeks' gestation. Of the remaining participants, we were able to calculate BRI for 9671 (96.3%) and BMI for 9812 (97.7%) of the eligible participants (Figure 1). Baseline demographic characteristics of included pregnancies are presented in Table 1. There were modest differences in maternal age and gestational age at visit 1 across early pregnancy BRI and BMI categories. However, we compared mean BRI and BMI between participants enrolled at 6 to 9 6/7 weeks and 10 to 13 6/7 weeks and did not observe differences by gestational age at study entry. With greater BRI and BMI categories, participants were more likely to self‐report non‐Hispanic Black race and Hispanic ethnicity. Those in the highest first trimester BRI and BMI categories were more likely to report current tobacco use and less likely to report some college education completion. These participants also had higher rates of publicly funded health insurance, pregestational diabetes, and chronic hypertension.

**TABLE 1:** Baseline characteristics by early pregnancy BRI and BMI category.

Percentile BRI/BMI values	Early pregnancy BRI categories (*n* = 9671)				*p*	Early pregnancy BMI categories (*n* = 9812)				*p*
	≤ 3rd (≤ 1.67), *n* = 292	4th–52nd (1.68–3.20), *n* = 4721	53rd–77th (3.21–4.45), *n* = 2405	≥ 78th (≥ 4.46), *n* = 2253		≤ 3rd (< 18.5), *n* = 230	4th–52nd (18.5–24.9), *n* = 4965	53rd–77th (25–29.9), *n* = 2444	≥ 78th (≥ 30), *n* = 2173	
Maternal age (years)	25.9 ± 5.5	27.2 ± 5.6	27.2 ± 5.7	26.6 ± 5.7	< 0.01	24.3 ± 5.6	27.2 ± 5.6	27.0 ± 5.7	26.7 ± 5.7	< 0.01
Gestational age at visit 1 (weeks)	11.3 ± 1.6	11.6 ± 1.5	11.6 ± 1.5	11.5 ± 1.6	< 0.01	11.5 ± 1.5	11.6 ± 1.5	11.6 ± 1.5	11.5 ± 1.6	0.74
Race and ethnicity (*n* = 9671)/(*n* = 9812)										
Non‐Hispanic White	171 (58.6)	3186 (67.5)	1390 (57.8)	1122 (49.8)	< 0.01	120 (52.2)	3251 (65.5)	1438 (58.8)	1115 (51.3)	< 0.01
Non‐Hispanic Black	49 (16.8)	481 (10.2)	317 (13.2)	480 (21.3)		33 (14.4)	488 (9.8)	344 (14.1)	512 (23.6)	
Hispanic	34 (11.6)	592 (12.5)	489 (20.3)	480 (21.3)		42 (18.3)	711 (14.3)	480 (19.6)	376 (17.3)	
Asian	19 (6.5)	246 (5.2)	89 (3.7)	43 (1.9)		23 (10)	281 (5.7)	65 (2.7)	30 (1.4)	
Other	19 (6.5)	216 (4.6)	120 (5)	128 (5.7)		12 (5.2)	234 (4.7)	117 (4.8)	140 (6.4)	
Education (*n* = 9671)/(*n* = 9812)										
High school or less	118 (40.4)	1516 (32.1)	960 (39.9)	1173 (52.1)	< 0.01	137 (59.6)	1605 (32.3)	986 (40.3)	1100 (50.6)	< 0.01
At least some college	174 (59.6)	3205 (67.9)	1445 (60.1)	1080 (47.9)		93 (40.4)	3360 (67.7)	1458 (59.7)	1173 (49.4)	
Insurance (*n* = 9609)/(*n* = 9749)										
Private	120 (41.2)	2571 (54.8)	1289 (54)	1100 (49.1)	< 0.01	105 (45.9)	2708 (54.9)	1275 (52.6)	1060 (49.1)	< 0.01
Public	79 (27.1)	1092 (23.3)	662 (27.7)	820 (36.6)		91 (39.7)	1162 (23.6)	686 (28.3)	777 (36)	
Other	92 (31.6)	1028 (21.9)	438 (18.3)	318 (14.2)		33 (14.4)	1065 (21.6)	465 (19.2)	322 (14.9)	
Current tobacco use (*n* = 9670)/(*n* = 9807)	44 (15.1)	711 (15.1)	416 (17.3)	547 (24.3)	< 0.01	43 (18.7)	743 (15)	438 (17.9)	528 (24.3)	< 0.01
Pregestational diabetes (*n* = 9158)/(*n* = 9285)	1 (0.4)	24 (0.5)	30 (1.3)	87 (4.1)	< 0.01	0	32 (0.7)	32 (1.4)	83 (4)	< 0.01
Chronic hypertension (*n* = 9151)/(*n* = 9278)	1 (0.4)	34 (0.8)	39 (1.7)	155 (7.3)	< 0.01	1 (0.5)	34 (0.7)	44 (1.9)	158 (7.7)	< 0.01
BMI at enrollment (*n* = 9657)/(*n* = 9812)										
Underweight	188 (64.4)	663 (14.1)	16 (0.7)	8 (0.4)	< 0.01	230 (100)	0	0	0	—
Normal weight	100 (34.2)	3424 (72.6)	670 (27.9)	61 (2.7)		0	4965 (100)	0	0	
Overweight	0	614 (13)	1363 (56.7)	434 (19.3)		0	0	2444 (100)	0	
Obesity	4 (1.4)	18 (0.4)	353 (14.7)	1741 (77.6)		0	0	0	2173 (94.6)	
BRI at enrollment (*n* = 9671)/(*n* = 9661)										
≤ 3 (< 18.5)	292 (100)	—	—	—		82 (36)	206 (4.2)	0	4 (0.2)	< 0.01
4–52 (18.5–24.9)	—	4721 (100)	—	—		140 (61.4)	3947 (80.4)	614 (25.5)	18 (0.8)	
53–77 (25–29.9)	—	—	2405 (100)	—		1 (0.4)	685 (14)	1363 (56.6)	353 (16.7)	
≥ 78% (≥ 30)	—	—	—	2253 (100)		5 (2.2)	69 (1.4)	433 (18)	1741 (82.3)	

*Note:* Data shown as *n* (%) or mean ± SD. Data complete except as noted.

Abbreviations: BMI, body mass index; BRI, body roundness index.

Table 1 demonstrates that there was incomplete concordance for the BRI and BMI quantiles. For individuals classified to an underweight BMI (Q1), 146 (64.0%) had a Q2 to Q4 category ranking in BRI (1.68 to ≥ 4.46). Among those classified to normal BMI category (Q2), 206 (4.2%) fell into Q1 for BRI (≤ 1.67), while 754 (15.4%) were assigned to Q3 and Q4 BRI (≥ 3.21). Among individuals with an overweight early pregnancy BMI (Q3), 614 (25.5%) were assigned to Q1 and Q2 BRI (< 3.21) and 433 (18%) to Q4 BRI (≥ 4.46). Lastly, among those with a BMI in the obesity category (Q4), 375 (17.7%) had a Q1 to Q3 BRI (< 4.46).

Table 2 illustrates the relationship between BRI, BMI, and adverse perinatal outcomes. Those in Q3 BRI and BMI had higher gestational weight gain. The likelihood of GDM, hypertensive disorders of pregnancy, and cesarean delivery increased across BRI and BMI categories. Rates of preterm birth were highest among those at the extremes of BRI (Q1, ≤ 1.67 and Q4, ≥ 4.46) and BMI (≥ 30 kg/m^2^). There were no differences in stillbirth rates by BRI or BMI categories. Rates of LGA birth weight were more frequent across BRI and BMI categories while those of SGA birth weight were less frequent.

**TABLE 2:** Perinatal outcomes by early pregnancy BRI and BMI quantiles.

Percentile BRI/BMI values	Early pregnancy BRI categories (*n* = 9671)				*p*	Early pregnancy BMI categories (*N* = 9812)				*p*
	≤ 3rd (≤ 1.67), *n* = 292	4th–52nd (1.68–3.20), *n* = 4721	53rd–77th (3.21–4.45), *n* = 2405	≥ 78th (≥ 4.46), *n* = 2253		≤ 3rd (< 18.5), *n* = 230	4th–52nd (18.5–24.9), *n* = 4965	53rd–77th (25–29.9), *n* = 2444	≥ 78th (≥ 30), *n* = 2173	
Weight gain (lb) (*n* = 8868)	31.9 ± 12.2	34.7 ± 13.5	35.5 ± 15.9	31.7 ± 21.5	< 0.01	29.7 ± 10.8	34.2 ± 13.0	36.3 ± 16.4	31.7 ± 22.1	< 0.01
Mean *z*‐score	2.5	2.8	3.1	2.9		2.1	2.7	3.2	3.0	
GDM (*n* = 9103)/(*n* = 9227)	8 (2.9)	89 (2)	112 (5)	173 (8.4)	< 0.01	8 (3.8)	113 (2.4)	96 (4.2)	169 (8.5)	< 0.01
Hypertensive disorders of pregnancy (*n* = 9149)/(*n* = 9276)	51 (18.8)	812 (18.1)	529 (23.3)	735 (34.5)	< 0.01	28 (13.3)	821 (17.4)	584 (25.5)	718 (34.9)	< 0.01
Preterm birth (*n* = 9334)/(*n* = 9462)	33 (11.9)	359 (7.9)	237 (10.2)	177 (12.7)	< 0.01	20 (9.2)	392 (8.2)	230 (9.8)	273 (13)	< 0.01
Stillbirth (*n* = 9671)/(*n* = 9812)	0	24 (0.5)	9 (0.4)	16 (0.7)	0.36	0	26 (0.5)	11 (0.5)	13 (0.6)	0.90
Cesarean delivery (*n* = 9159)/(*n* = 9286)	42 (15.5)	916 (20.4)	682 (30)	884 (41.5)	< 0.01	31 (14.8)	966 (20.5)	717 (31.2)	852 (41.4)	< 0.01
Birth weight category (*n* = 9126)/(*n* = 9252)										
AGA	224 (82.7)	3703 (82.8)	1843 (81.6)	1662 (78.2)		163 (77.3)	3896 (82.8)	1857 (81.3)	1614 (78.7)	
SGA	39 (14.4)	504 (11.3)	228 (10.1)	210 (9.9)	< 0.01	43 (20.4)	548 (11.7)	228 (10)	188 (9.2)	< 0.01
LGA	8 (3)	263 (5.9)	187 (8.3)	252 (11.9)		5 (2.4)	261 (5.5)	199 (8.7)	250 (12.2)	

*Note:* Data shown as *n* (%) or mean ± SD. Data complete except as noted.

Abbreviations: AGA, appropriate for gestational age; BRI, body roundness index; GDM, gestational diabetes; LGA, large for gestational age; SGA, small for gestational age.

**FIGURE 2:**
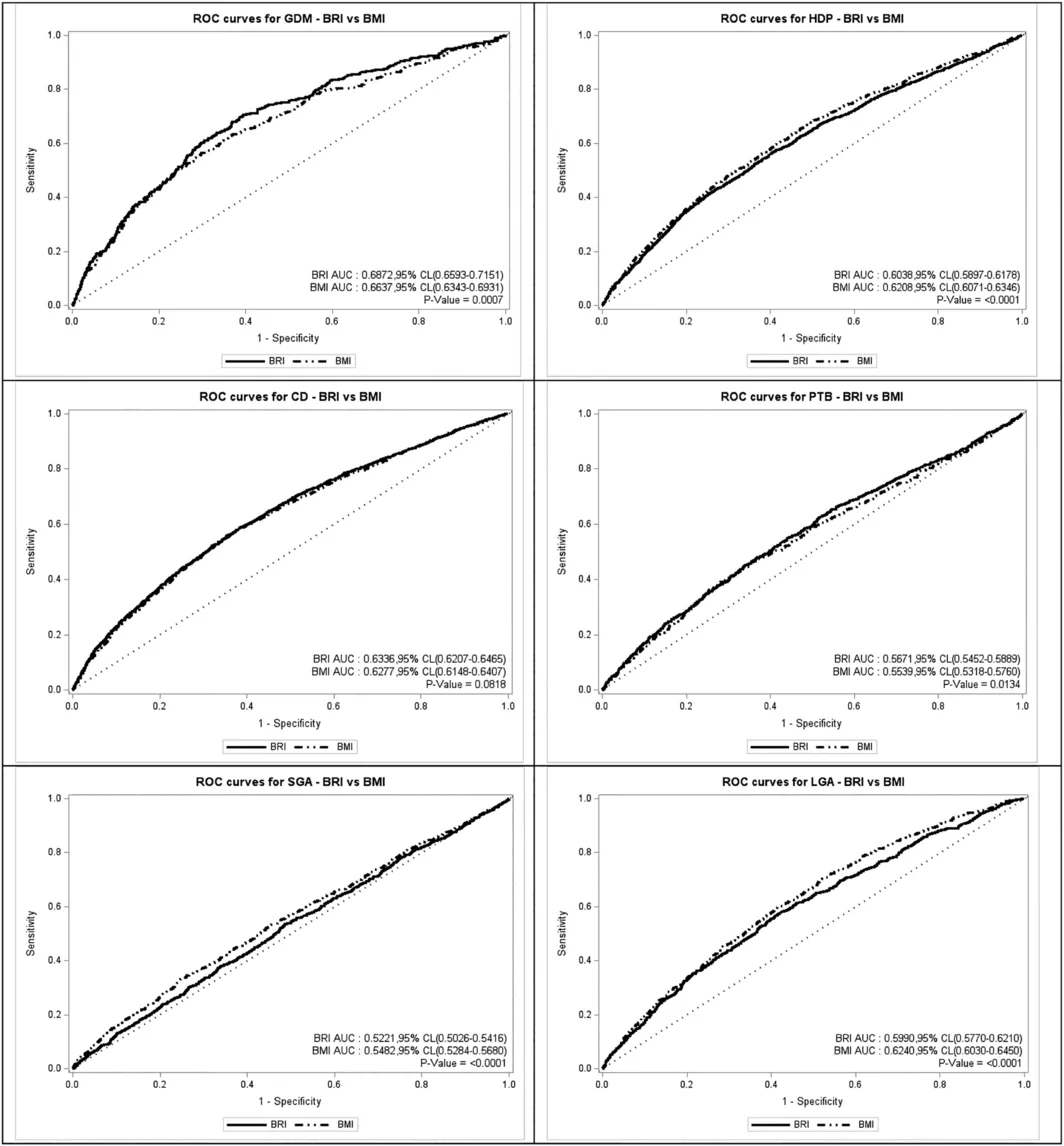
Comparison of receiver operating characteristic (ROC) curves for the association of BMI and BRI with adverse pregnancy outcomes. BMI and BRI were each fitted as continuous variables in separate logistic regression models. Models adjusted for age, maternal race, smoking, insurance type, pregestational diabetes, and chronic hypertension.

As shown in Table 3, using Q2 BRI (1.68–3.20) and BMI (normal, 18.5–24.9) categories as the reference and adjusting for covariates, both GDM and hypertensive disorders of pregnancy were more likely to occur among individuals with a Q4 BRI (≥ 4.46) or BMI (≥ 30 kg/m^2^). Preterm birth was more likely among all compared BRI quantiles (Q1, Q3, Q4) and Q4 BMI (≥ 30 kg/m^2^). The risk for cesarean delivery increased in Q3 and Q4 for both BRI (≥ 3.21) and BMI (≥ 25 kg/m^2^). LGA birth weight was more common in those with a Q3 and Q4 BRI (≥ 3.21) and BMI (≥ 25 kg/m^2^) while SGA birth weight was less common. Having a Q1 BMI (< 18.5 kg/m^2^) was associated with an increased likelihood of SGA birth weight. We compared AUCs for the fully adjusted models of BRI and BMI for each of the adverse outcomes with the results shown in Figure 2. Overall, BRI and BMI had similar discriminatory capacities for each outcome. BRI performed better for GDM (AUC 0.687, *p* = 0.0007) and trended toward improved performance for cesarean delivery but did not achieve significance (*p* = 0.08). BMI better distinguished pregnancies resulting in LGA birth weight (AUC 0.624, *p* < 0.0001) and receiving a diagnosis of hypertensive disorders of pregnancy (AUC 0.621, *p* < 0.0001).

**TABLE 3:** Adjusted associations between early pregnancy BRI and BMI and pregnancy outcomes.

Percentile BRI/BMI values	Early pregnancy BRI categories				Early pregnancy BMI categories			
	≤ 3rd (≤ 1.67)	4th–52nd (1.68–3.20)	53rd–77th (3.21–4.45)	≥ 78th (≥ 4.46)	≤ 3rd (< 18.5)	4th–52nd (18.5–24.9)	53rd–77th (25–29.9)	≥ 78th (≥ 30)
GDM	1.49 (0.71–3.12)	Ref	2.63 (1.97–3.50)	4.75 (3.61–6.24)	1.50 (0.72–3.14)	Ref	1.89 (1.42–2.50)	4.14 (3.20–5.36)
Hypertensive disorders of pregnancy	0.96 (0.70–1.31)	Ref	1.42 (1.25–1.61)	2.43 (2.15–2.75)	0.74 (0.49–1.11)	Ref	1.65 (1.46–1.87)	2.54 (2.25–2.88)
Preterm birth < 37 weeks	1.54 (1.02–2.37)	Ref	1.27 (1.05–1.53)	1.37 (1.14–1.66)	1.08 (0.65–1.80)	Ref	1.12 (0.93–1.34)	1.32 (1.10–1.59)
Cesarean delivery	0.73 (0.52–1.02)	Ref	1.65 (1.46–1.85)	2.63 (2.34–2.87)	0.68 (0.46–1.00)	Ref	1.74 (1.55–1.95)	2.66 (2.37–3.00)
Birth weight category								
SGA	1.23 (0.86–1.75)	Ref	0.84 (0.71–0.99)	0.73 (0.61–0.88)	1.78 (1.25–2.53)	Ref	0.80 (0.68–0.95)	0.64 (0.53–0.77)
LGA	0.52 (0.26–1.07)	Ref	1.47 (1.21–1.79)	2.20 (1.82–2.66)	0.45 (0.18–1.09)	Ref	1.66 (1.36–2.01)	2.41 (1.99–2.91)

*Note:* Data shown as odds ratio with 95% CI. Models adjusted for age, maternal race, smoking, insurance type, pregestational diabetes, and chronic hypertension.

Abbreviations: BRI, body roundness index; GDM, gestational diabetes; LGA, large for gestational age; SGA, small for gestational age.

We next conducted a stratified analysis evaluating the relationship between discordant BRI and BMI on adverse pregnancy outcomes as shown in Table 4. In women with a normal BMI (Q2, 18.5–24.9 kg/m^2^), a higher BRI quantile assignment (Q3 to Q4, ≥ 3.21; BRI > BMI) was associated with an increase in the risk for GDM (aOR 2.28, 95% CI 1.49–3.45) and cesarean delivery (aOR 1.39, 95% CI 1.15–1.68). In contrast, those with a lower BRI group (Q1, ≤ 1.67; BRI < BMI) experienced an increased risk for preterm birth (aOR 1.64, 95% CI 1.05–2.55). For women with an overweight BMI (Q3, 25–29.9 kg/m^2^), a lower BRI quantile (Q1 to Q2, < 3.21; BRI < BMI) was associated with a significantly lower risk for GDM (aOR 0.31, 95% CI 0.15–0.63). Among this same cohort of participants, there was a decreased risk for cesarean delivery (aOR 0.75, 95% CI 0.60–0.94). Lastly, participants with an obesity category BMI (Q4, ≥ 30 kg/m^2^) but lower BRI category (Q1 to Q3, < 4.46; BRI < BMI) experienced reduced risk for cesarean delivery (aOR 0.55, 95% CI 0.43–0.70) and hypertensive disorders of pregnancy (aOR 0.63, 95% CI 0.49–0.81). There were no differences in rates of SGA or LGA birth weight across BMI categories regardless of the BRI category.

**TABLE 4:** Stratified analysis of discordant early pregnancy BRI and BMI classification and pregnancy outcomes.

Variable	BRI and BMI classification			BRI < BMI vs. BRI = BMI		BRI > BMI vs. BRI = BMI	
	BRI < BMI	BRI = BMI	BRI > BMI	OR (95% CI)	*p*	OR (95% CI)	*p*
BMI underweight (*n* = 292)	*n* = 0	*n* = 82	*n* = 146				
GDM	—	4 (5.3)	4 (2.9)	—	—	0.54 (0.13–2.22)	0.39
HDP	—	13 (17.8)	13 (9.6)	—	—	0.49 (0.22–1.13)	0.09
PTB	—	9 (11.5)	11 (8)	—	—	0.66 (0.26–1.68)	0.39
Cesarean delivery	—	8 (11)	23 (17)	—	—	1.67 (0.71–3.95)	0.24
SGA	—	15 (20.6)	27 (19.9)	—	—	0.96 (0.47–1.94)	0.90
LGA	—	1 (1.4)	4 (2.9)	—	—	2.18 (0.24–19.89)	0.49
BMI normal (*n* = 4721)	*n* = 206	*n* = 3947	*n* = 754				
GDM	4 (2.1)	76 (2)	32 (4.5%)	1.02 (0.37–2.81)	0.97	**2.28 (1.49–3.47)**	**< 0.01**
HDP	38 (19.6)	656 (17.5)	120 (16.8%)	1.15 (0.80–1.66)	0.45	0.96 (0.77–1.18)	0.67
PTB	24 (12.2)	300 (7.9)	66 (9.1%)	**1.64 (1.05–2.55)**	**0.03**	1.17 (0.89–1.55)	0.26
Cesarean delivery	34 (17.5)	739 (19.6)	181 (25.4%)	0.87 (0.60–1.27)	0.47	**1.39 (1.15–1.68)**	**< 0.01**
SGA	24 (12.4)	416 (11.1)	94 (13.3%)	1.13 (0.73–1.76)	0.58	1.23 (0.96–1.56)	0.10
LGA	7 (3.6)	209 (5.6)	44 (6.2%)	0.63 (0.30–1.37)	0.25	1.12 (0.80–1.57)	0.50
BMI overweight (*n* = 2444)	*n* = 613	*n* = 1363	*n* = 434				
GDM	9 (1.6)	62 (4.9)	24 (5.9)	**0.31 (0.15–0.63)**	**< 0.01**	1.23 (0.76–2.00)	0.40
HDP	138 (24.2)	329 (25.8)	109 (26.3)	0.92 (0.73–1.15)	0.46	1.03 (0.80–1.32)	0.85
PTB	48 (8.1)	131 (10)	49 (11.6)	0.80 (0.56–1.13)	0.20	1.18 (0.83–1.67)	0.35
Cesarean delivery	149 (26.1)	408 (31.9)	147 (35.4)	**0.75 (0.60–0.94)**	**0.01**	1.17 (0.93–1.48)	0.19
SGA	58 (10.2)	116 (9.1)	47 (11.4)	1.13 (0.81–1.57)	0.48	1.27 (0.89–1.82)	0.19
LGA	50 (8.8)	110 (8.7)	39 (9.4)	1.02 (0.72–1.44)	0.93	1.10 (0.75–1.61)	0.64
BMI obesity (*N* = 2173)	*n* = 375	*n* = 1741	*n* = 0				
GDM	22 (6.2)	145 (9.1)	—	0.66 (0.41–1.05)	0.08	—	—
HDP	97 (27)	611 (37.2)	—	**0.63 (0.49–0.81)**	**< 0.01**	—	—
PTB	42 (11.4)	223 (13.2)	—	0.85 (0.60–1.20)	0.35	—	—
Cesarean delivery	108 (30.1)	723 (44)	—	**0.55 (0.43–0.70)**	**< 0.01**	—	—
SGA	28 (7.8)	155 (9.5)	—	0.81 (0.54–1.24)	0.34	—	—
LGA	36 (10.1)	210 (12.8)	—	0.76 (0.52–1.11)	0.15	—	—

*Note:* Data shown as *n* (%) or odds ratio (OR) with 95% CI. Data shown in bold were identified as statistically significant. Models adjusted for age, maternal race, smoking, insurance type, pregestational diabetes, and chronic hypertension.

Abbreviations: GDM, gestational diabetes; HDP, hypertensive disorders of pregnancy; LGA, large for gestational age; PTB, preterm birth; SGA, small for gestational age.

## 4 Discussion

We found that greater adiposity, as evidenced by both first trimester BRI and BMI, is associated with multiple adverse pregnancy outcomes, including GDM, hypertensive disorders of pregnancy, cesarean delivery, preterm birth, and LGA birth weight in a dose‐dependent fashion. Like BMI, early pregnancy BRI overall identified similar patterns of risk and exhibited fair discriminatory ability for GDM, hypertensive disorders of pregnancy, and cesarean delivery. Despite these similarities, our ROC and exploratory stratified analyses suggest that BRI may have an edge over BMI for GDM and cesarean delivery risk stratification, possibly due to its improved capacity to characterize weight distribution. Our analysis reaffirms that waist circumference and adiposity distribution within BMI categories are variable [25]. In fact, nearly half of the participants with a normal BMI had a discordant BRI quantile assignment with the majority in BRI Q3 and Q4. BRI exerted similar modifications on risk for GDM and cesarean delivery in the normal and overweight BMI groups, but in opposite ways: a higher BRI quantile than BMI increased risk in those with a normal BMI, while a lower quantile BRI than BMI decreased risk in those with a BMI indicating overweight. Interestingly, the converse was not true. While discordance was less frequent in the participants with a BMI indicating obesity, a lower corresponding BRI quantile (Q1 to Q3) again drove down risk of cesarean delivery and hypertensive disorders of pregnancy. These discrepancies support that BRI adds value to understanding the impact of weight on pregnancy outcomes, likely for some groups more than others, such as those with a normal BMI.

To date, there are limited data evaluating the relationship between BRI and adverse pregnancy outcomes. Moreover, prior studies have used varied gestational ages for measurement, which impacts an index dependent upon waist circumference. Shi et al. previously identified a 39% increase in GDM per BRI unit rise. However, this was the only outcome examined by the authors, and the timing of BRI measurement in relation to the GDM diagnosis was unclear. In contrast, our analysis used a larger dataset with rigorously and prospectively collected pregnancy outcomes, minimizing ascertainment bias [14]. BMI is a widely accepted, easily measured, and validated tool for assessing weight risk in pregnancy [4], and the relationship between BMI and adverse pregnancy outcomes is well characterized [26, 27]. Our findings suggest both GDM and cesarean delivery may be more sensitive to the distribution of central adiposity rather than weight alone. Higher BRI has been associated with diabetes development in non‐pregnant adults and GDM in pregnant adults [8, 14]. Pregnancy itself increases insulin resistance by approximately 40%–50% [28], which may be compounded by pre‐existing central adiposity. Central adiposity is thought to arise from increased visceral fat, leading to insulin resistance and β‐cell dysfunction. Visceral fat is metabolically active, releasing hormones, cytokines, such as TNF‐a, and free fatty acids that interfere with the body's response to insulin and promote inflammation [29]. In a pregnant rat model, surgical removal of visceral adipose tissue decreased hepatic insulin resistance and triglycerides to prepregnancy levels despite late gestation [30], highlighting the active nature of visceral adiposity. Additional animal studies illustrate negative effects of early pregnancy insulin resistance and hyperglycemia exposure to the fetoplacental unit, such as excess glycogen deposition, reactive oxygen species, and impaired decidual function [28]. These biologic mechanisms not only reinforce the risk for GDM, but also suggest pathways for placental damage and malperfusion. Placental inflammation and injury can lead to antiangiogenic factor release, increasing the risk for preeclampsia and decreasing placental reserve to mitigate the stress of labor, potentially leading to cesarean delivery [31]. Central adiposity has also been hypothesized to increase risk for cesarean delivery because excess adipose tissue in the abdomen and pelvis can obstruct labor and limit fetal descent. BMI ≥ 30 kg/m^2^ is linked to slower cervical ripening and uterine contractions, as well as decreased sensitivity to oxytocin. Lastly, obesity is a risk factor for LGA birth weight, amplifying the risk for cesarean delivery due to cephalopelvic disproportion [32, 33].

A systematic review and meta‐analysis by Heslehurst et al. [13] compiled 70 studies consisting of nearly 90,000 participants to evaluate the utility of anthropometric indices in pregnancy. The authors found that higher waist circumference was associated with increased odds of developing both GDM and hypertensive disorders of pregnancy. Although this study robustly collates available evidence, there was significant heterogeneity among studies, such as waist circumference measurements up to 20 weeks' gestation. In addition, this meta‐analysis did not address additional clinically meaningful perinatal outcomes linked to obesity, including mode of delivery and extremes of birth weight. Lastly, this analysis did not compare the anthropometric indices to BMI performance. Our analysis of a prospective cohort using first trimester waist circumference measures allows us to comprehensively assess the relationship between BRI and perinatal outcomes.

Diagnosing obesity has significant social and medical implications, and elevated BMI is often associated with an “unhealthy” state [34]. However, in non‐pregnant adults, BMI has low sensitivity and does not identify a significant proportion of those with excess body fat [35]. Appreciable proportions of individuals from the NHANES database with overweight and obesity were metabolically healthy (29% of those with overweight BMI and 16% of those with obesity BMI), while 30% of individuals with a normal BMI were metabolically unhealthy using blood pressure, lipid, glucose, insulin resistance, and C‐reactive protein measures [36]. In a Canadian cohort study of hundreds of thousands of pregnancies, 60% of pregnant patients with obesity did not experience obstetric complications [37]. This is highlighted by our ROC analyses, which show poor or fair discriminatory capacities of both early pregnancy BMI and BRI in association with adverse pregnancy outcomes. These discrepancies suggest additional anthropometric tools are needed. In non‐pregnant adults, waist circumference has a strong association with diabetes, hypertension, dyslipidemia, and mortality [7, 25, 38]. BRI may have advantages in estimating visceral body fat because it also accounts for height [6]. Greater BRI is significantly associated with adverse cardiometabolic outcomes and mortality [7, 12, 32] and has outperformed BMI and waist circumference in some studies [39]. Notably, waist circumference is not routinely obtained during prenatal care at present. The Lancet Diabetes and Endocrinology Commission proposed a recent redefinition of obesity. For those with BMI > 40 kg/m^2^, excess body fat is pragmatically assumed. However, an obesity diagnosis in those with BMI between 30 and 39.9 kg/m^2^ requires a second anthropometric measure to confirm excess body fat (e.g., waist circumference). If obesity is confirmed, “preclinical” and “clinical” obesity are differentiated by the presence of obesity‐related organ‐system dysfunction or limitations to daily activities [5]. Pregnant individuals with elevated BMI may not have weight‐related organ dysfunction or activity impairment due to their relatively young age to make this diagnostic distinction. However, the physiologic changes of pregnancy in conjunction with excess baseline maternal adiposity may predispose to conditions such as GDM, which are associated with significant lifelong risks for type 2 diabetes, cardiovascular disease, and metabolic risks to offspring [28]. Improved characterization of obesity in pregnancy may allow providers to optimize surveillance and weight management, as well as capture patients outside the extremes of weight but with excess abdominal adiposity.

Strengths of our study include a large, diverse cohort with waist circumference obtained in a standard fashion by trained research staff and perinatal outcomes carefully assessed prospectively. However, our analysis was limited by lack of consensus definitions of “normal” and “abnormal” BRI values and limits the external validity of the results. To address this, we intentionally compared BRI to BMI using similar categories to inform current clinical practice and the broader discussion about whether BMI or BRI is a better measure for adverse outcomes. As this is an observational study, we are limited in the ability to account for every possible confounder. Although waist circumference was obtained at study entry between 6 and 13 weeks and 6 days of gestation and is unlikely to be affected by the growing uterus at this early gestational age, our findings may not be generalizable to non‐pregnant individuals or those beyond the first trimester. Expanding on this, the parent study enrolled only nulliparas limiting the generalizability to all pregnant individuals. Parity may affect waist circumference; however, it appears this difference is minimized after adjusting for confounding variables, such as BMI and age [40]. Lastly, these findings will require validation in an independent cohort.

## 5 Conclusion

In this large, diverse cohort of nulliparous pregnant patients in the US, greater BRI was associated with significantly increased rates of adverse pregnancy outcomes. Although BRI and BMI were associated with similar increased risks for adverse outcomes, BRI may help further refine risk for some complications such as GDM and cesarean delivery. Further work is needed to see if this anthropometric measure can inform management strategies in pregnancy and to assess whether it is associated with long‐term metabolic risks in pregnant individuals.

## Author Contributions

C.M.S. and K.J.P. researched data and wrote the first draft of the manuscript. S.B. and Y.T. completed the statistical analysis and reviewed and edited the manuscript. M.N.F., D.M.H., T.M.B., U.M.R., R.M.S., and L.M.Y. reviewed and edited the manuscript. All authors approved the final version of the manuscript. C.M.S. is the guarantor of this work and, as such, had full access to all the data in the study and takes responsibility for the integrity of the data and the accuracy of the data analysis.

## Funding

Research reported in this publication was supported by the Eunice Kennedy Shriver National Institute of Child Health and Human Development of the National Institutes of Health under institution specific award numbers (U10 HD063036, RTI International; U10 HD063072, Case Western Reserve University; U10 HD063047, Columbia University; U10 HD063037, Indiana University; U10 HD063041, University of Pittsburgh; U10 HD063020, Northwestern University; U10 HD063046, University of California Irvine; U10 HD063048, University of Pennsylvania; and U10 HD063053, University of Utah). The content is solely the responsibility of the authors and does not necessarily represent the official views of the National Institutes of Health.

## Conflicts of Interest

C.M.S. has previously consulted for Visterra and Otsuka Pharmaceuticals. The remaining authors declare no conflicts of interest.

## Data Availability

The data that support the findings of this study are available from the corresponding author upon reasonable request.
